# New-Onset Gambling Addiction Following Treatment for Psychosis With Cariprazine

**DOI:** 10.7759/cureus.97709

**Published:** 2025-11-24

**Authors:** Muneeb Hashmi, Angel Nguyen, Hibah Ilyas

**Affiliations:** 1 Naresh K. Vashisht College of Medicine, Texas A&M University, Bryan, USA; 2 John Sealy School of Medicine, University of Texas Medical Branch at Galveston, Galveston, USA; 3 Psychiatry, Valley Health System, Las Vegas, USA

**Keywords:** addiction, aripiprazole, atypical antipsychotics, cariprazine, compulsive gambling, iatrogenic complication, impulse control disorder, psychosis

## Abstract

Cariprazine is an atypical antipsychotic primarily used to treat schizophrenia and bipolar I disorder. Unlike most antipsychotics, it is effective in managing both the positive and negative symptoms of schizophrenia. However, it has recently been associated with impulse control disorders (ICDs) as a potential side effect. This case report describes a 42-year-old female patient with schizoaffective disorder who developed new-onset compulsive gambling following initiation of cariprazine therapy, despite well-controlled psychotic symptoms. After discontinuing cariprazine, the patient experienced a notable improvement in impulse control with complete cessation of gambling behavior. This report highlights the importance of careful monitoring for such side effects in patients receiving cariprazine and, to our knowledge, is the first to describe compulsive gambling associated with this medication.

## Introduction

Cariprazine is an atypical antipsychotic primarily prescribed for the treatment of schizophrenia and bipolar disorder. It functions as a partial agonist at dopamine D2 and D3 receptors, with a higher affinity for D3 receptors, and also exhibits partial agonism at serotonin 5-HT1A receptors. This receptor profile contributes to its efficacy in managing both positive and negative symptoms of schizophrenia, as well as manic or mixed episodes associated with bipolar I disorder.

While cariprazine is generally well-tolerated, some patients may experience side effects, including akathisia, insomnia, and gastrointestinal disturbances. Notably, there have been reports linking cariprazine to impulse control disorders (ICDs), which the Diagnostic and Statistical Manual of Mental Disorders, 5th Edition (DSM-5) defines as “the failure to resist an impulse, drive, or temptation to perform an act that is harmful to the person or to others" [1]. These behaviors are thought to arise from cariprazine’s partial agonism at D3 receptors, which are implicated in the brain's reward pathways [2].

Pharmacovigilance data further illustrate the clinical relevance of this issue. A 2021 analysis of the EudraVigilance database identified a strong association between cariprazine and ICDs, with a reporting odds ratio of 28.3 [3]. However, this signal was based on just seven reports, suggesting that ICDs remain a relatively uncommon adverse effect of cariprazine. When antipsychotic-induced ICDs do emerge, management typically involves discontinuation or dose reduction of the offending agent; thus, early recognition is crucial in mitigating the psychosocial consequences of these behaviors [4].

In this case report, we present a patient who developed new-onset gambling behavior following the initiation of cariprazine therapy. Upon discontinuation of cariprazine, the patient's gambling behavior ceased.

This case underscores the importance of monitoring for impulse control issues in patients receiving cariprazine and highlights the potential for these behaviors to resolve following the discontinuation of the medication, emphasizing the need for individualized treatment strategies in managing complex psychiatric conditions. To our knowledge, this is the first case report describing compulsive gambling behavior associated with cariprazine use, though a case of hypersexuality has previously been documented.

## Case presentation

The patient is a 42-year-old, married Southeast Asian female. She had first been hospitalized at an inpatient psychiatric facility at the age of 38 for a depressive episode, but did not adhere to her prescribed medication regimen after discharge. A few years later, local law enforcement brought her to the same hospital following reports from her family of bizarre behavior. Her family stated that she had been urinating in a trash bag and locking herself and her eight-year-old daughter in her room. They also reported that she had been starving herself and her daughter. On evaluation, the patient appeared paranoid and verbalized delusional beliefs that her husband was harming her daughter. She was evasive yet cooperative during the encounter. She denied suicidal or homicidal ideations as well as other mood, psychotic, or anxiety symptoms but demonstrated poor judgment and insight, appearing unaware of her symptoms and the need for psychiatric treatment.

Initially diagnosed with an unspecified psychotic disorder, she was admitted for inpatient care for seven days, during which she complied with cognitive behavioral therapy and a regimen of aripiprazole (Abilify), 5 mg daily. Following stabilization, she was transitioned to partial hospitalization, where she was additionally diagnosed with hypertension and prescribed lisinopril, 10 mg daily. Aripiprazole was also increased to 10 mg daily. She remained compliant with her medications, demonstrated improved coping skills, and was no longer considered unsafe outside structured settings. Following discharge, she continued to receive treatment at a private mental health clinic. Over the next six months, the patient no longer expressed paranoid ideas, and her husband reported significant improvement in her well-being and daily functioning.

Three years later, she presented to the clinic again, accompanied by her husband. Since her previous visit, she had been taken off aripiprazole after experiencing a 40-pound weight gain, which was suspected to be a side effect of the medication. Her primary care physician instead prescribed cariprazine (Vraylar), 3 mg daily, to manage psychotic symptoms. This change in treatment occurred within the year preceding her visit, although the precise timeline is unclear. While her psychosis remained well-controlled, her husband expressed concern over a newly developed gambling addiction. Despite no prior history of impulsive behaviors, the patient had been compulsively playing online slot machines for the past eight weeks. This escalated to the point that their bank account had gone into the negative, yet she was unable to control these behaviors. She was subsequently diagnosed with schizoaffective disorder and ICD and prescribed fluoxetine (Prozac), 20 mg daily, to address compulsive gambling. Additionally, due to concerns that her gambling behavior was a potential side effect of cariprazine, it was discontinued, and she was prescribed xanomeline and trospium chloride (Cobenfy), 50 mg/20 mg twice daily. Upon her follow-up visit two weeks later, the patient reported improvements in impulse control, stating that she felt more in control of her urges to gamble. She had not experienced a recurrence of psychotic symptoms and continued to function well at home.

## Discussion

Cariprazine (Vraylar) is an atypical, third-generation antipsychotic that acts as a partial agonist at dopamine D2 and D3 receptors, as well as serotonin 5-HT1A receptors. It also functions as an antagonist at serotonin 5-HT1A and histamine H1 receptors. While other D2 partial agonist antipsychotics primarily aim to treat the positive symptoms of schizophrenia, cariprazine's tenfold higher affinity for D3 over D2 receptors contributes to its unique effectiveness in addressing negative symptoms [2]. It also exhibits antidepressant properties, likely associated with its activity at 5-HT1A receptors [5]. As such, cariprazine is FDA-approved for the treatment of schizophrenia, bipolar I disorder, and adjunctive therapy for major depressive disorder.

According to the DSM-5, ICDs are characterized by the “failure to resist an impulse, drive, or temptation to perform an act that is harmful to the person or to others” and include behaviors such as hypersexuality, compulsive shopping, pathological gambling, and binge eating [1]. Although the literature exploring cariprazine's association with ICDs is limited, a pharmacovigilance case/non-case study found a strong link between ICDs and cariprazine, with a reporting odds ratio (ROR) of 28.3 when compared to all drugs (n = 7) [3]. However, this association disappeared when compared specifically to other antipsychotics, such as aripiprazole and brexpiprazole, which are both partial D2 agonists with lower D3 affinity and have a well-documented, stronger link to ICDs [1,3]. Aripiprazole in particular has demonstrated a dose-related risk of ICDs, which often resolve with dose reduction or discontinuation [1,3]. In contrast, cariprazine has shown a much lower and less well-established risk for ICDs in the available literature; thus, it is particularly noteworthy that the patient described in this case developed impulse control issues with cariprazine but not aripiprazole.

A separate case report describes a 67-year-old Caucasian female with schizophrenia who developed hypersexuality seven days after initiation of cariprazine, which resolved upon discontinuation [6]. In both cases, symptoms emerged at a dosage of 3 mg, and the absence of prior tendencies toward impulsivity strengthens the plausibility of a causal relationship.

Similar concerns have been raised about other dopamine D3-preferring agents. For example, pramipexole, a D3 agonist used to treat symptoms of Parkinson's disease, has been associated with a significant number of ICD cases due to its high D3 receptor selectivity [7]. Interestingly, a rat study found that D1 receptor activity in the nucleus accumbens supported adaptive, risk-based decision-making, while D3 receptor activation was associated with reduced risk-taking and reward sensitivity [8]. These findings suggest a nuanced role for D3 receptors in modulating decision-making and reward-related behavior. Chronic cariprazine treatment in rats has also been associated with a reduction in dopaminergic neurons in the ventral tegmental area, suggesting potential desensitization effects from prolonged treatment [9]. This supports the idea that cariprazine can modulate dopaminergic signaling in reward-related brain areas, which may underlie both its therapeutic and side effect profiles.

**Figure 1:**
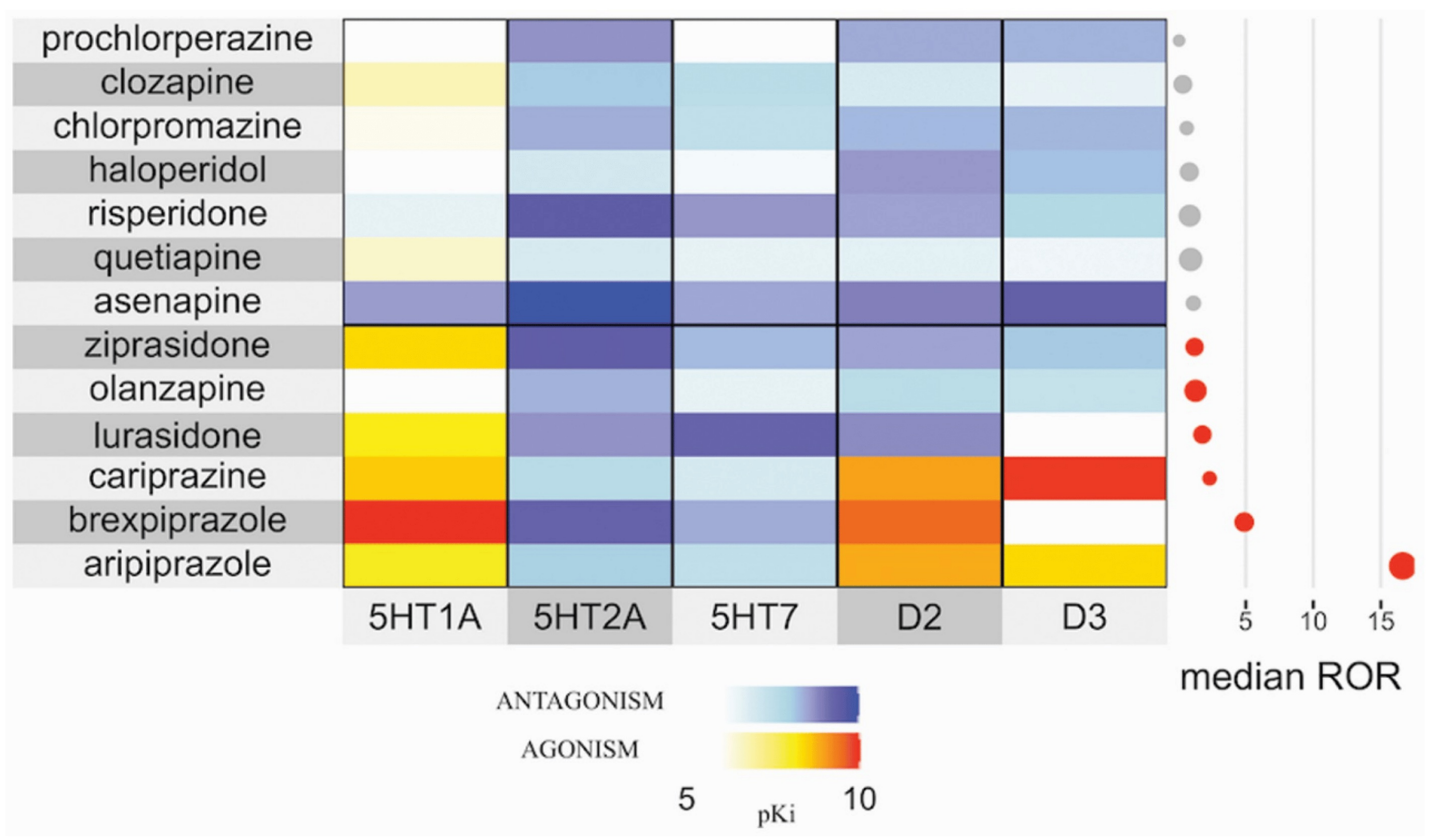
Receptor Binding Profiles of Antipsychotics and Their Potential Association With Impulse Control Disorders 5HT1A/2A/7: serotonin 1A/2A/7 receptors; D2/3: dopamine 2/3 receptors; pKi: measure of a drug's binding affinity to a receptor; ROR: reporting odds ratio; ICD: impulse control disorder. Warmer colors indicate greater receptor agonism, cooler colors indicate antagonism, and the adjacent plot displays significant (red) and non-significant (gray) median RORs of ICDs. Cariprazine shows a notable association with ICDs. Figure republished from [10] under a Creative Commons Attribution (CC BY 4.0) license, which permits unrestricted reuse, distribution, and reproduction in any medium, provided the original work is properly cited.

As discussed earlier, the risk of ICDs appears much lower with cariprazine when compared to other third-generation antipsychotics like aripiprazole. Still, pharmacovigilance data from the FDA Adverse Event Reporting System (FAERS) indicate that cariprazine may show a detectable signal for ICDs when compared to all drugs, as illustrated in Figure 1 [10]. Given the growing body of evidence linking D3 receptor activity to risky behaviors, clinicians should exercise caution when prescribing cariprazine to patients with a history of impulse control issues. Standardized tools such as the Questionnaire for Impulsive-Compulsive Disorders in Parkinson's Disease (QUIP) and the Barratt Impulsiveness Scale (BIS-11), recommended by the American Academy of Neurology, can help identify behaviors associated with dopamine agonist use and ICDs [11].

Additionally, genetic variants in dopamine receptor genes (e.g., DRD2 and DRD3) may influence receptor sensitivity and individual susceptibility to ICDs, as seen in carriers of the DRD3 Ser9Gly (rs6280) CT genotype [12]. Future research into these polymorphisms could enhance our ability to predict and manage ICD risk in patients treated with cariprazine.

Certain limitations to this report should be noted. As a single case, the findings have limited generalizability, and causality cannot be definitively established. Although the patient’s compulsive gambling resolved following discontinuation of cariprazine, and she reportedly had no prior history of impulsive behaviors, a clearer timeline of cariprazine initiation relative to the onset of gambling would strengthen the possible correlation. Given these limitations, we further evaluated the likelihood of a drug-related effect using the Naranjo Adverse Drug Reaction Probability Scale, which produced a score of 5, suggesting probable causality [13].

While cariprazine’s D3 receptor activity makes it an effective agent for negative symptoms and mood disorders, its potential association with ICDs warrants further investigation. Clinicians should remain cautious and consider individual risk factors when initiating treatment.

## Conclusions

This report highlights the resolution of compulsive gambling behavior following the discontinuation of cariprazine in a 42-year-old female patient with schizoaffective disorder and ICD. It underscores the importance of monitoring for impulse control disturbances as a possible iatrogenic effect of cariprazine, as such conditions related to partial dopamine agonists can easily be overlooked or mistaken for progression of the underlying psychiatric illness. Clinician and patient education regarding this potential side effect is crucial to facilitate early recognition and intervention. Additionally, routine screening for new-onset compulsive behaviors should be incorporated into follow-up visits for patients receiving cariprazine or other partial dopamine agonists such as aripiprazole and brexpiprazole. Future reports of similar adverse effects associated with this class of antipsychotics will strengthen the evidence base, improving clinical awareness and informing safer treatment approaches.
